# An anatomic aberration and a surgical challenge: Mediastinal parathyroid adenoma anterior the pericardium. A case report

**DOI:** 10.1016/j.ijscr.2019.04.005

**Published:** 2019-04-06

**Authors:** Antonios Patrinos, Maria Zarokosta, Theodoros Piperos, John Tsiaoussis, George Noussios, Theodoros Mariolis-Sapsakos

**Affiliations:** a Anatomy and Histology Laboratory, School of Nursing, University of Athens, Greece; b University Department of Surgery, General and Oncologic Hospital of Kifissia “Agii Anargiri’’, Athens, Greece; c University Department of Anatomy, Faculty of Medicine, University of Crete, Greece; d Department of Anatomy, School of Physical Education and Sport Sciences, Serres, Aristotles University of Thessaloniki, Greece

**Keywords:** Single parathyroid adenoma, Aberrant location, PHPT, Hypercalcemia, Case report

## Abstract

**Introduction:**

Ectopic parathyroid glands occur in 6–16% of cases of PHPT and they constitute a potential cause of failed primary surgical therapy. In particular, aberrant adenomas located deeper in the mediastinum, as in the presented case, remain a severe challenge for the surgeons.

**Presentation of case:**

A 54-year-old Caucasian female proceeded to our institution with signs and symptoms of PHPT. Imaging studies performed identified a large mass localized in the lower anterior mediastinum, on the left of the median line. A mid-sternal thoracotomy was performed and the aberrant adenoma was finally detected anterior to the pericardium and the left pericardiophrenic vessels and the left phrenic nerve. The operation was uneventful. A meticulous review of the literature was conducted as well.

**Discussion:**

Single parathyroid adenomas are the key culprits of PHPT. Anatomic aberrations of the location of the parathyroid glands and their adenomas are more common than described in the literature and there are possible anatomic aberrations that have not been described yet. All these anatomic variations constitute major risk-factors of thoracic bleeding and of nerve injury.

**Conclusion:**

Detailed preoperative detection in addition to meticulous exposure of the operative field are fundamental in order to perform a safe adenoma excision without harmful impacts to the patient.

## Highlights

• Ectopic parathyroid adenomas located deeper in the mediastinum remain a surgical challenge.
• Their incidence reaches up to 20% of the general population and they tend to constitute a severe cause of failed primary surgery for PHPT.
• Such aberrations seem to be more common than described in the literature and there are possible anatomic aberrations that have not been described yet.
• Preoperative detection of the mediastinal parathyroid adenoma and detailed exposure of the operative field are essential for a safe mid-sternal thoracotomy.

## 1 Introduction

Single parathyroid adenomas are the key culprits of primary hyperparathyroidism (PHPT) and therefore their preoperative localization is of paramount surgical importance [1,2]. Parathyroid glands and their adenomas are typically located on the posterior surface of the thyroid gland [3]. However, due to their embryology, they are predisposed to ectopic locations along the median line of the thorax and the neck, from the angle of the mandible to the mediastinum [3,4]. Mediastinal ectopic glands are typically detected in the superior mediastinum, into the thymus gland and they may be excised via a cervical incision [3]. However, ectopic adenomas located deeper in the mediastinum, as in the presented case, remain a surgical challenge [3]. The incidence of ectopic parathyroid glands reaches up to 20% of the general population [2] and their adenomas tend to constitute a severe cause of failed primary surgery for PHPT [4]. Hence, it is crucial to pinpoint precisely these tissues so as the patient undergo a successful operation. The present manuscript that aims to highlight a peculiar ectopic location of a parathyroid adenoma into the anterior mediastinum and to underline the importance of preoperative detection of ectopic parathyroid adenomas for the successful surgical treatment of rare mediastinal adenomas, has been reported in line with the SCARE criteria [5].

## 2 Case report

A 54-year-old Caucasian female proceeded to our institution with epigastric pain, nausea and vomiting along with pain located around the lumbar area lasting for one week. No previous surgical history or commorbidities existed. Clinical examination did not reveal any palpable abdominal masses or abdominal tenderness and the patient’s vital signs were within the normal spectrum. Blood test detected hypercalcemia (serum calcium: 10.2 mg/dL) and parathyroid hormone level of 111.8 pg/mL. All the findings in conjunction with the clinical presentation lead to the assumption that the patient had primary hyperparathyroidism (PHPT).

**Fig. 1:**
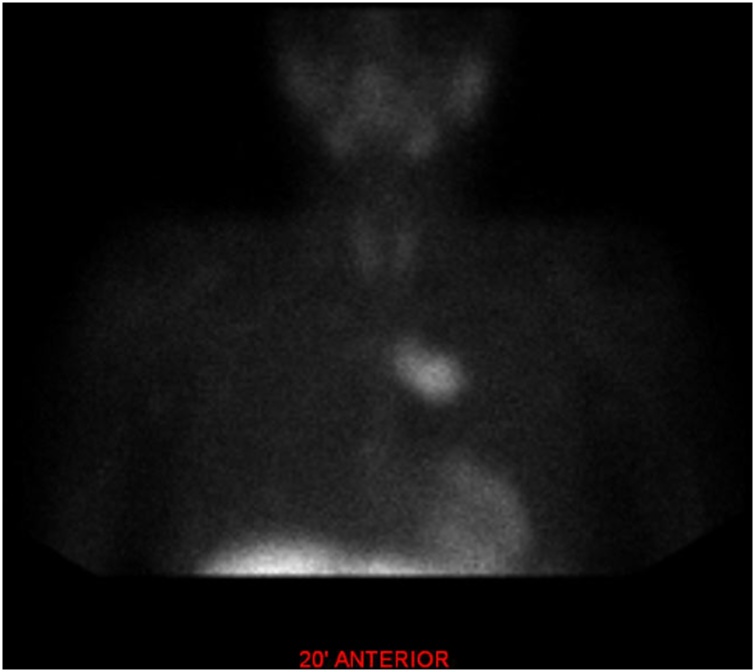
Tc-99m-MIBI scintigraphy detected an ectopic adenoma located in the lower anterior mediastinum, on the left of the median line.

Then, an ultrasound was performed but it was negative for any thyroid or parathyroid abnormalities. Subsequently, the thoracic and abdominal CT revealed a soft tissue in the anterior mediastinum 7 × 1 cm. Additional Tc-99m-MIBI scintigraphy followed, which detected an ectopic adenoma located in the lower anterior mediastinum, on the left of the median line (Fig. 1). Following these, a mid-sternal thoracotomy was finally scheduled.

**Fig. 2:**
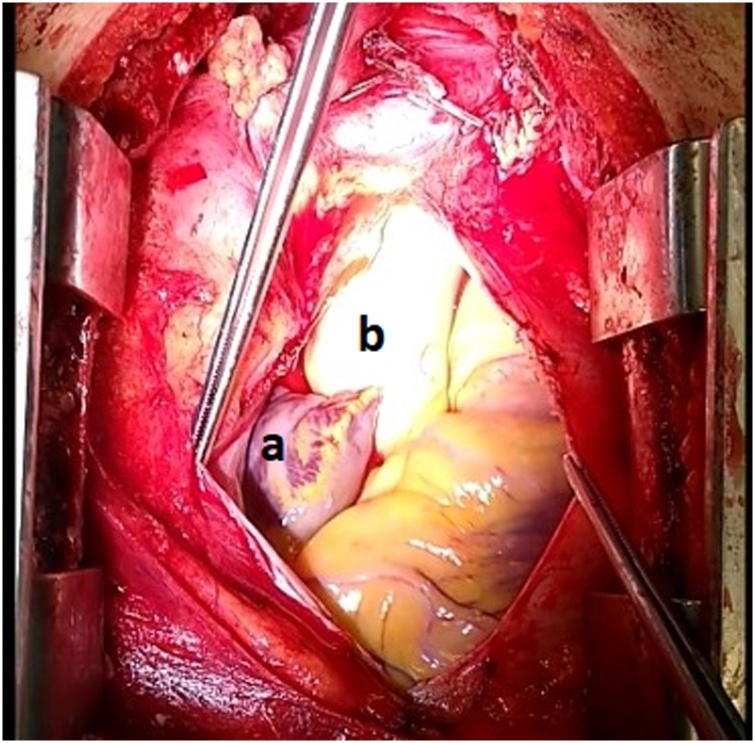
The operative field-meticulous view of the lower anterior mediastinum (a: aberrant mediastinal adenoma/ b: pericardiac sac).

**Figs. 3 and 4:**
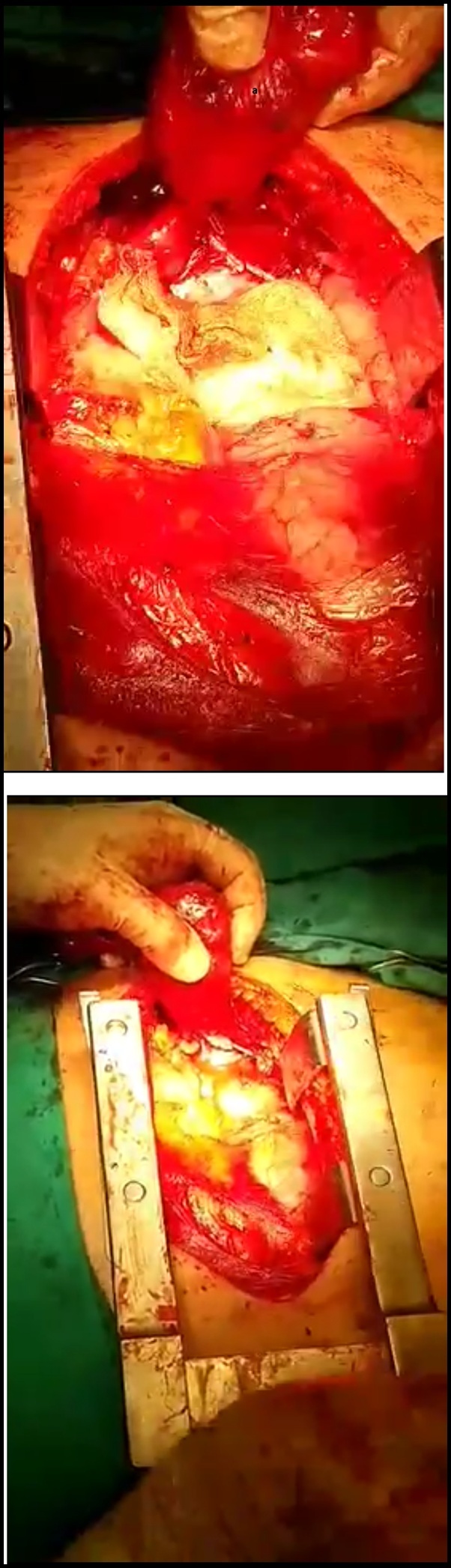
Gentle excision of the mediastinal adenoma from the thoracic cavity (a: parathyroid adenoma)..

During the operation, after the thoracotomy, surgeons attempted to detect deep into the mediastinum the parathyroid adenoma according to the preoparative localization. Indeed, the mediastinal mass was detected on the left of the median line, at the anterior mediastinum, in front of the anterior surface of the pericardium and close to the left pericardiophrenic vessels and the left phrenic nerve (Fig. 2). The adenoma was covered by a thin fibrous capsule. When surgeons removed the capsule, a dark red mass of 7 × 2.8 × 1 cm was finally revealed (Figs. 3 and 4 ). The detailful preoperative localization of the present mediastinal adenoma which was in close relation with various anatomical structures of the thorax, reduced effectively the difficulty of the mass excision and the potentiality of accidental surgical injuries which may lead to thoracic bleeding and subsequent obstructive symptoms.

Then, the operation continued in the usual fashion and a drainage was placed into the left side of the thoracic cavity. The patient was discharged the 5th postoperative day with instructions, when the drainage was finally removed.

Histology of the mass confirmed the diagnosis of ectopic parathyroid adenoma that was composed predominantly of oxyphil cells arranged in an acinar pattern. Serum calcium level was 2.60 mmol/L and iPTH 17.6 pg/mL 12 h after the operation. Serum calcium and iPTH remained normal after 6 months’ follow-up.

## 3 Discussion

Primary hyperparathyroidism (PHPT) affects approximately 25 per 100,000 in the general population and it is more prevalent among older people, aged 50-years old or more, as in the presented case [6,7]. The diagnosis of PHPT may be established when the patient presents hypercalcemia, hypophosphatemia and raised levels of alkaline phosphatase and iPTH and typical clinical manifestations, such as recurrent presence of kidney stones, hypertension, peptic ulcers and osteopenia [6,8,9].

PHPT is caused by the presence of a single adenoma (75–85%), of a carcinoma (1%) or multiple adenomas (4–5%) and due to hyperplasia of the parathyroid glands (10–20%) as well [10]. However, single parathyroid adenomas are the key culprits of PHPT and since they tend to present great variability concerning their location, their preoperative localization is of paramount surgical and clinical importance [1,2,11].

Ectopic parathyroid glands have been firstly reported in 1932 by Churchil [3] and according to the current literature they are detected in 6–16% of cases of PHPT [12] and their aberrant location is found along the median line of the thorax and the neck, from the angle of the mandible to the mediastinum [3,4]. Embryologically, the parathyroid glands are derived from the third and fourth pharyngeal pouches and descend along with the thyroid gland. In case of failure of the present process, the parathyroid glands may be detected in the neck posterior to the mandible or within the mediastinum as ectopic tissues [7,8].

The reported prevalence of ectopic parathyroid glands reaches up to 20% of the general population [2] and their ectopic adenomas tend to constitute a severe cause of failed primary surgery for PHPT [4]. Thus, it is mandatory and crucial to pinpoint precisely these tissues so as the patient undergo a successful operation, especially when encountering a mediastinal adenoma. Indeed, although the majority of ectopic glands, is found in the superior mediastinum and can be effectively excised via a cervical incision, mediastinal parathyroid adenomas that are detected deeper in the mediastinum, as in the presented case, remain a surgical challenge [3].

Ectopic parathyroid adenomas are often detected either in the preoperative work up or in post parathyroidectomy patients with persistent hypercalcemia, as the localization of an ectopic parathyroid adenoma may be challenging [8]. Nevertheless, the main concern of physicians in these rare cases is to spare patients suffering from PHPT from unnecessary interventions. For this reason, preoperative imaging interventions are essential before any exploration for parathyroid adenomas – in our case deep into the anterior mediastinum [7,13,14].

However, there is some controversy when it comes to choosing the optimal preoperative imaging techniques [3,12,15]. According to previous studies and case reports, ultrasonography did not aid in detecting mediastinal adenomas, as demonstrated in the presented case as well [7,14,15]. In addition, CT and MRI seem to contribute to identifying these adenomas and they tend to provide valuable information as for their anatomical correlation to nearby tissues [7,8,14,15]. Other studies show that a combination of CT and PET-CT could facilitate even more the localization of these adenomas [7,15]. Finally, as for Tc-99m-MIBI scintigraphy, its sensitivity (approximately 80–90%) showcases the importance of this modality for the detailful localization of ectopic parathyroid adenomas [7,14].

In the presented case, technetium sestamibi imaging was used in conjunction with CT for the establishment of the diagnosis of PHPT due to a large ectopic mediastinal parathyroid adenoma and for the prompt, preoperative planning of the safe surgical excision of the adenoma.

Conclusively, there are several documented ectopic locations of single parathyroid adenomas into the mediastinum [[1], [2], [3],[12], [13], [14], [15]]. Nevertheless, it seems that such aberrations are more common than described in the literature and that there are possible anatomic aberrations that have not been described yet. All these anatomic variations constitute major risk-factors of thoracic bleeding and of nerve injury, as the left phrenic nerve in the presented case [3]. Herein, prompt preoperative detection in addition to gentle and detailed exposure of the operative field are fundamental in order to perform a safe adenoma excision without harmful impacts to the patient.

## 4 Conclusions

Imaging modalities are of paramount clinical importance in preoperative diagnostic steps of ectopic parathyroid adenomas. In fact, combination of several techniques, as in the presented case, may provide better localization of the ectopic adenoma and detection of its relationship with adjacent anatomical structures. Hence, meticulous preoperative detection of the mediastinal parathyroid adenoma and detailed exposure of the operative field is the cornerstone of a safe mid-sternal thoracotomy in addition to surgeons’ perpetual awareness concerning this scarce anatomical aberration.

## Conflicts of interest

All authors declare that there are not any competing interests.

## Funding

There is no source of funding.

## Ethical approval

This is a Case Report for which the patient provided written informed consent. Ethical approval has also been provided by the ethical committee of the General & Oncologic Hospital of Kifissia “Agii Anarguri”.

## Consent

Written consent for the publication of this case report and accompanying images was obtained from the patient. The consent can be provided to the Editor if he asks so. The written approval of the Ethical Committee of our Institution may be provided to the Editor as well.

## Author contribution

Mariolis-Sapsakos and Patrinos conceived of the study. Tsiaoussis and Noussios were senior consultants at this case report and participated in its coordination. Patrinos and Zarokosta contributed to literature review. Patrinos, Zarokosta and Piperos contributed to the preparation of the manuscript. Noussios, Mariolis-Sapsakos and Tsiaoussis contributed to the refinement of the case report. All authors have approved the final article.

## Registration of research studies

This is a Case Report and according to the Research Registry, its registration is not essential.

## Guarantor

Theodoros Mariolis-Sapsakos.

## Provenance and peer review

Not commissioned, externally peer-reviewed.
